# Crystal structure and Hirshfeld surface analysis of 4-methyl-*N*-[2-(5-methyl­furan-2-yl)phen­yl]-*N*-[(5-phenyl­furan-2-yl)meth­yl]benzene­sulfonamide

**DOI:** 10.1107/S2056989025006231

**Published:** 2025-07-17

**Authors:** Selbi Annadurdyyeva, Anastasia E. Levina, Victor N. Khrustalev, Roya Z. Nazarova, Khudayar I. Hasanov, Nurlana D. Sadikhova, Mehmet Akkurt, Gizachew Mulugeta Manahelohe

**Affiliations:** a RUDN University, 6 Miklukho-Maklaya St., Moscow 117198, Russian Federation; b Zelinsky Institute of Organic Chemistry of RAS, 4, 7 Leninsky Prospect, 119991 Moscow, Russian Federation; c Baku Engineering University, Khirdalan, Hasan Aliyev str. 120, AZ0101, Absheron, Azerbaijan; d Azerbaijan Medical University, Scientific Research Centre (SRC), A. Kasumzade St. 14, AZ 1022, Baku, Azerbaijan; e Department of Organic Chemistry, Baku State University, Z. Xalilov Str. 23, AZ 1148 Baku, Azerbaijan; f Department of Physics, Faculty of Sciences, Erciyes University, 38039 Kayseri, Türkiye; g Department of Chemistry, University of Gondar, PO Box 196, Gondar, Ethiopia; Universidade Federal do ABC, Brazil

**Keywords:** crystal structure, hydrogen bond, van der Waals inter­actions, Hirshfeld surface analysis

## Abstract

In the title compound, C_29_H_25_NO_4_S, the mol­ecular conformation is stable with intra­molecular C—H⋯O and C—H⋯N inter­actions being observed. In the crystal, mol­ecules are linked by C—H⋯O inter­actions, forming layers parallel to the (100) plane. In addition, π–π [centroid-to-centroid distance = 3.4961 (7) Å] and C—H⋯π inter­actions connect the mol­ecules within the layers. The layers are also bound to each other by van der Waals inter­actions. A Hirshfeld surface analysis of the crystal structure indicates that the most important contributions for the crystal packing are from H⋯H (53.2%), C⋯H/H⋯C (28.9%) and O⋯H/H⋯O (13.8%) contacts.

## Abstract 2

In the crystal, mol­ecules are linked by C—H⋯O inter­actions, forming layers parallel to the (100) plane. In addition, π–π [centroid-to-centroid distance = 3.4961 (7) Å] and C—H⋯π inter­actions connect mol­ecules within the layers. The layers are also bound to each other by van der Waals inter­actions.

## 1. Chemical context

Carbon–carbon bond formation *via* Suzuki coupling of organo­boronic acids and its derivates with organic halides provides a mild method for the synthesis of various functionalized compounds (Miyaura & Suzuki, 1995 ▸; Suzuki 1999 ▸), especially bi­aryls (Leadbeater & Marco, 2002 ▸; Polyanskii *et al.*, 2019 ▸; Khalilov *et al.*, 2021 ▸). Recently, we have been inter­ested in the synthesis of furan-substituted sulfonyl­amides because they are key inter­mediates in synthesis, analytical chemistry, catalysis and in the pharmaceutical industry (Demeke & Forsyth, 2002 ▸; Alieva *et al.*, 2005 ▸; Aliyeva *et al.*, 2024 ▸). The most widely used furan-substituted sulfonyl­amide is furosemide, which is a loop diuretic medication used to treat fluid build-up due to heart failure, kidney disease, or liver scarring. Continuing our research in the chemistry of furyl-substituted sulfonamides (Guliyeva *et al.*, 2024 ▸; Mammadova *et al.*, 2023**a* ▸,b* ▸; Burkin *et al.*, 2024 ▸, 2025 ▸), in this work we set out a coupling strategy using the Suzuki reaction to synthesize a sulfonyl­amide-substituted bi­furan compound, which was hitherto unknown and is potentially important toward further transformations or the synthesis of pharmaceutical species. Moreover, the attachment of non-covalent bond-donor or acceptor centers to the sulfonyl­amide can be applied as a synthetic strategy in the ligand design and catalysis (Gurbanov *et al.*, 2022 ▸; Huseynov *et al.*, 2021 ▸; Mahmudov *et al.*, 2015 ▸, 2023 ▸).
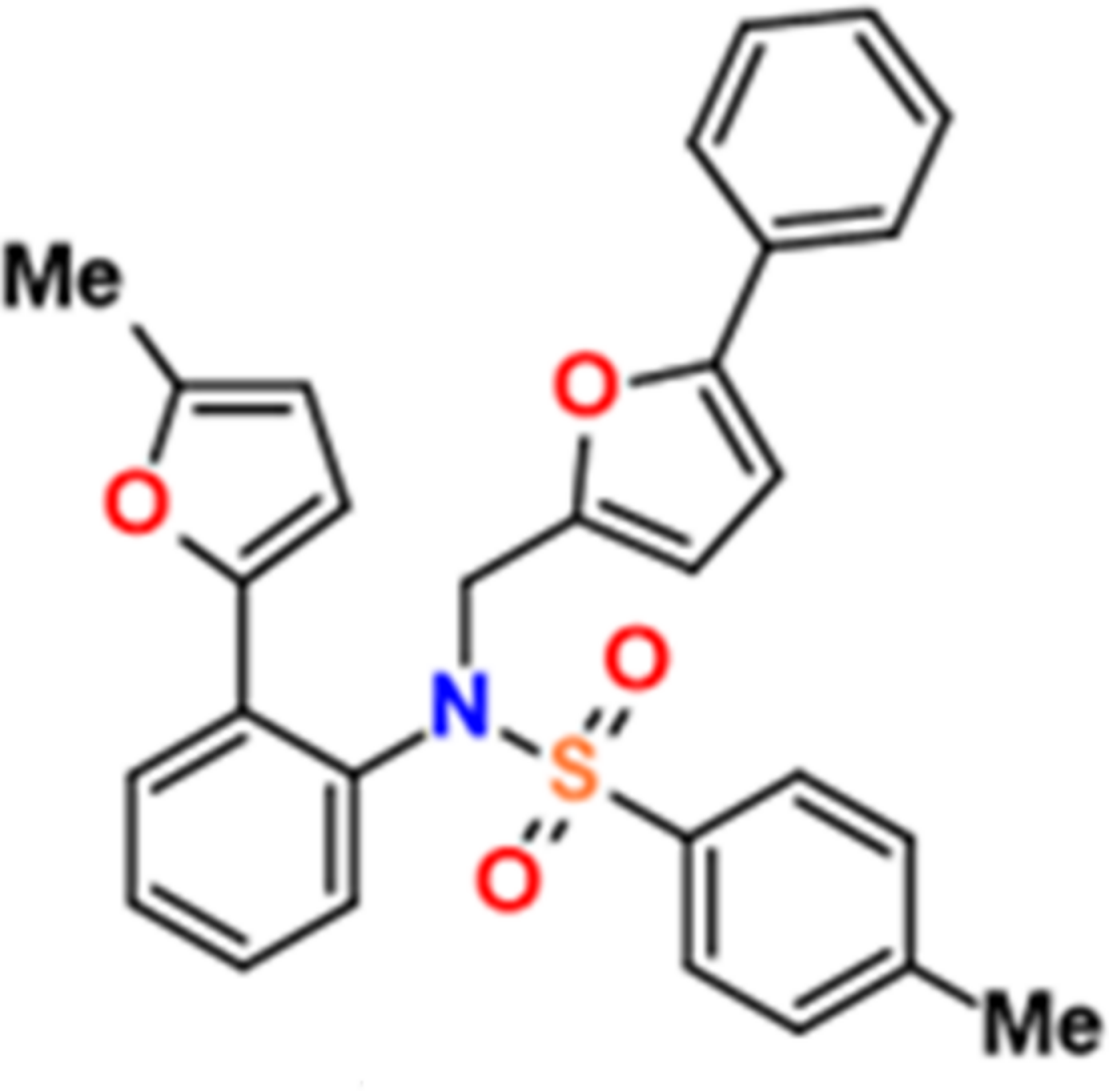


## 2. Structural commentary

**Figure 1:**
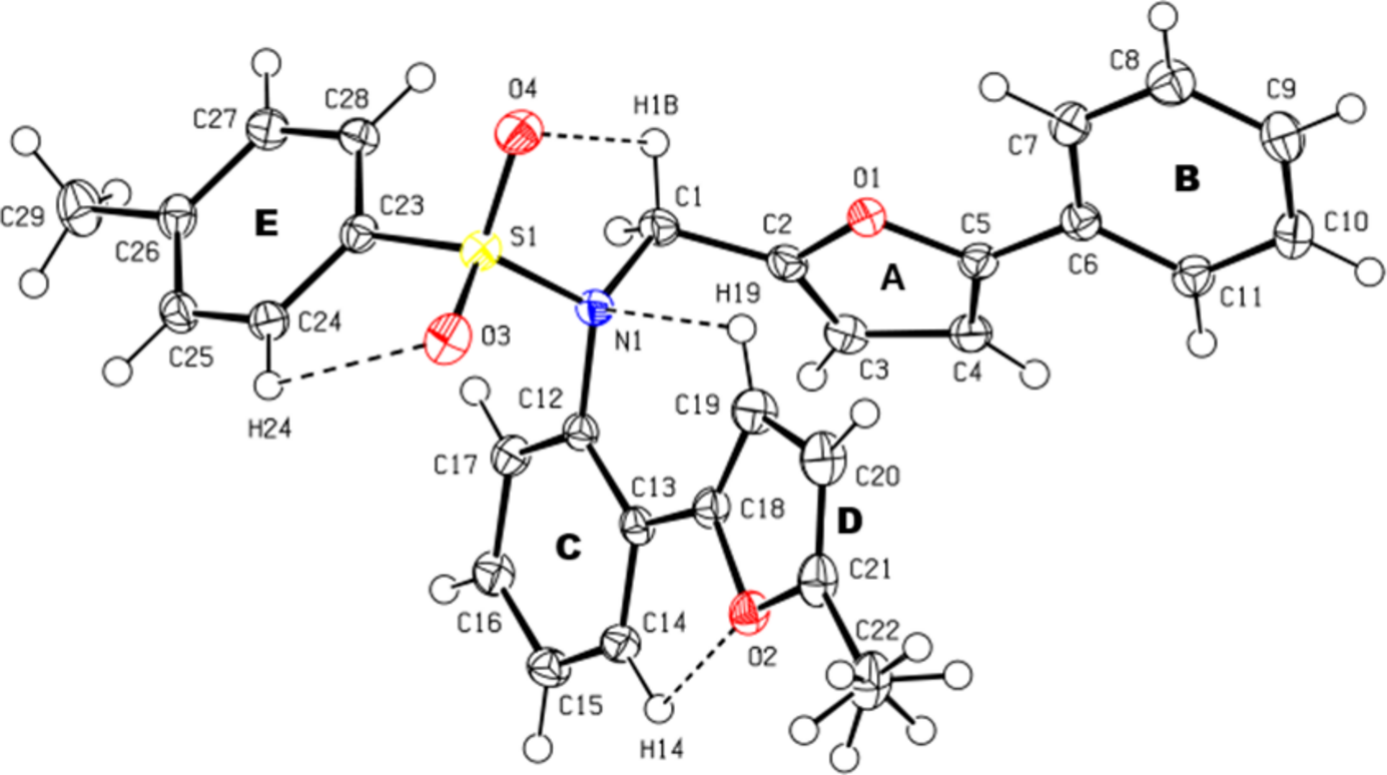
View of the title mol­ecule. Displacement ellipsoids are drawn at the 50% probability level.

The mol­ecular conformation of the mol­ecule is stable due to the intra­molecular inter­actions C1—H1*B*⋯O4, C14—H14⋯O2, C19—H19⋯N1 and C24—H24⋯O3, which form *S*(5), *S*(5), *S*(6) and *S*(5) ring motifs (Bernstein *et al.*, 1995 ▸), respectively (Fig. 1 ▸; Table 1 ▸). The dihedral angles between the planes of the *A* (O1/C2–C5), *B* (C6–C11), *C* (C12–C17), *D* (O2/C18–C21), and *E* (C23–C28) rings of the mol­ecule are *A*/*B* = 13.26 (7), *A*/*C* = 52.20 (7), *A*/*D* = 62.45 (8), *A*/*E* = 88.06 (7), *B*/*C* = 48.74 (6), *B*/*D*= 53.59 (7), *B*/*E* = 75.63 (6), *C*/*D* = 5.41 (7), *C*/*E* = 39.61 (6) and *D*/*E* =35.00 (7)°. The bond lengths and angles in the title compound are in good agreement with those reported for related compounds (see *Database survey* section).

## 3. Supra­molecular features and Hirshfeld surface analysis

**Figure 2:**
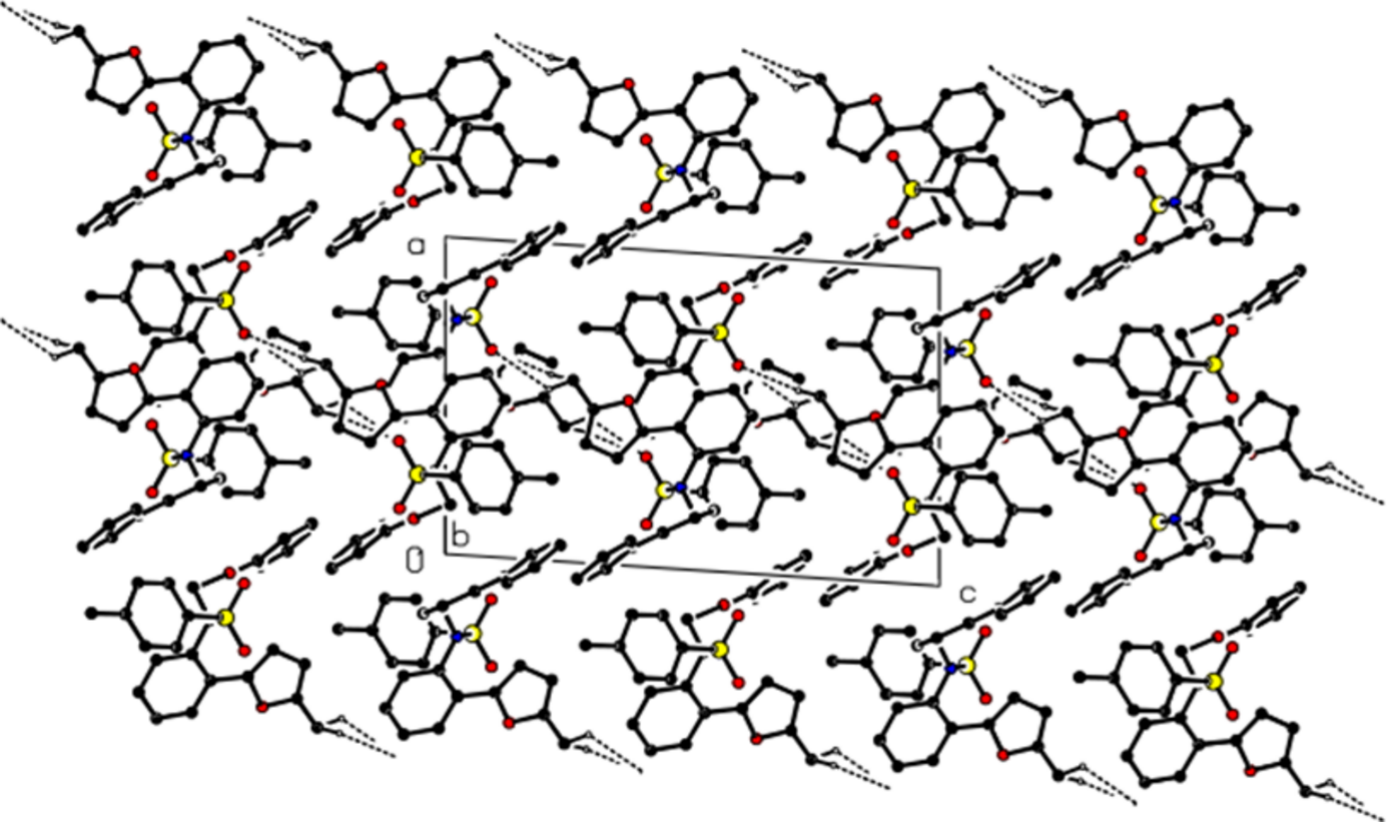
A view of the mol­ecular packing along the *b* axis, showing the C—H⋯O inter­actions.

**Figure 4:**
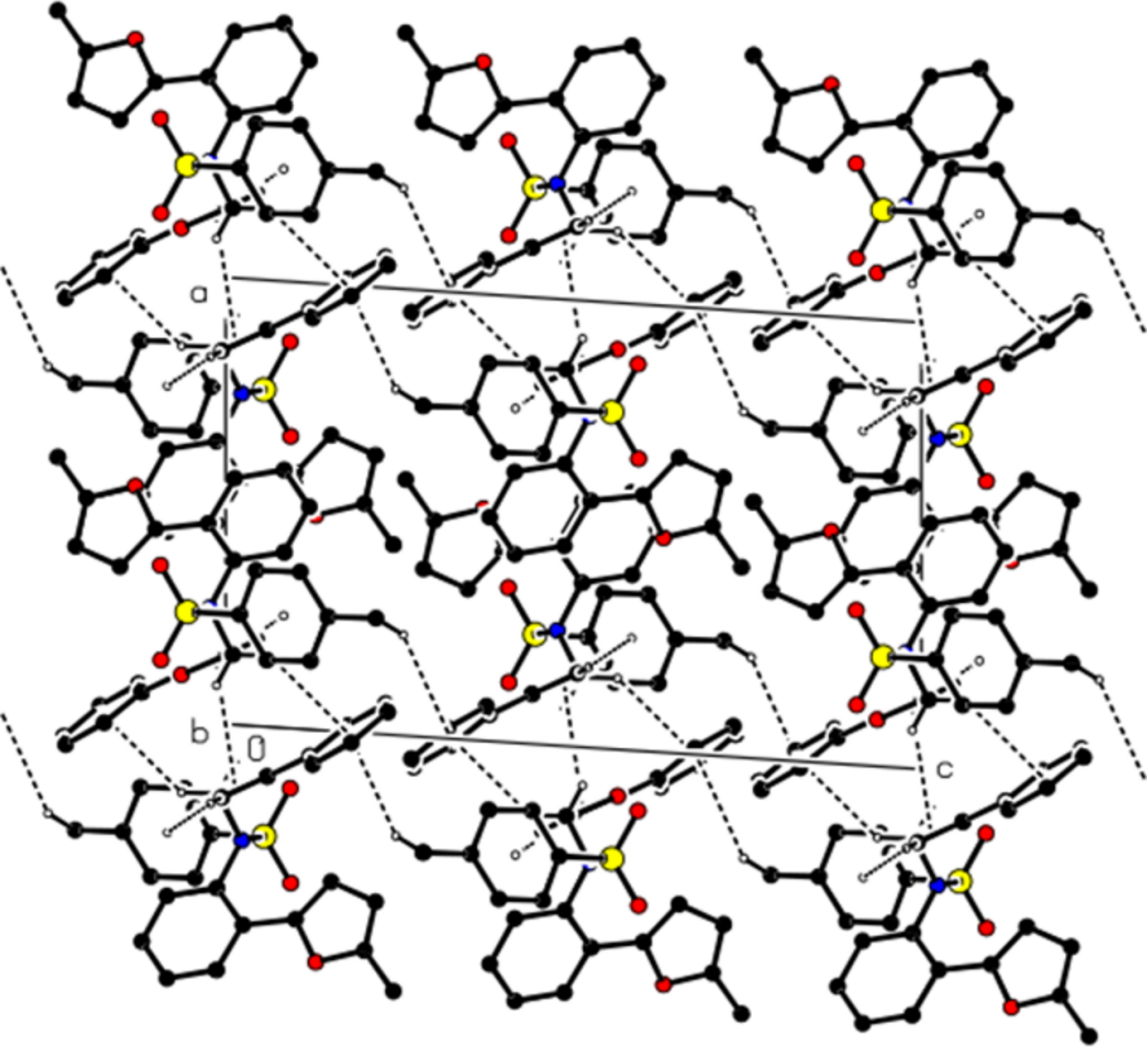
A view of the mol­ecular packing along the *b* axis, showing the π–π and C—H⋯π inter­actions.

In the crystal, mol­ecules are linked by the inter­molecular C—H⋯O inter­actions, forming layers parallel to the (100) plane (Table 1 ▸; Figs. 2 ▸ and 3 ▸). In addition, π–π [*Cg*1⋯*Cg*1^ii^ = 3.4961 (7) Å, slippage = 1.016 Å; symmetry code: (ii) −*x* + 2, −*y* + 1, −*z* + 1; *Cg*1 is the centroid of the (*A*: O1/C2–C5) furan ring] and C—H⋯π inter­actions connect the mol­ecules within layers (Fig. 4 ▸). The layers are also bound to each other by van der Waals inter­actions.

**Figure 5:**
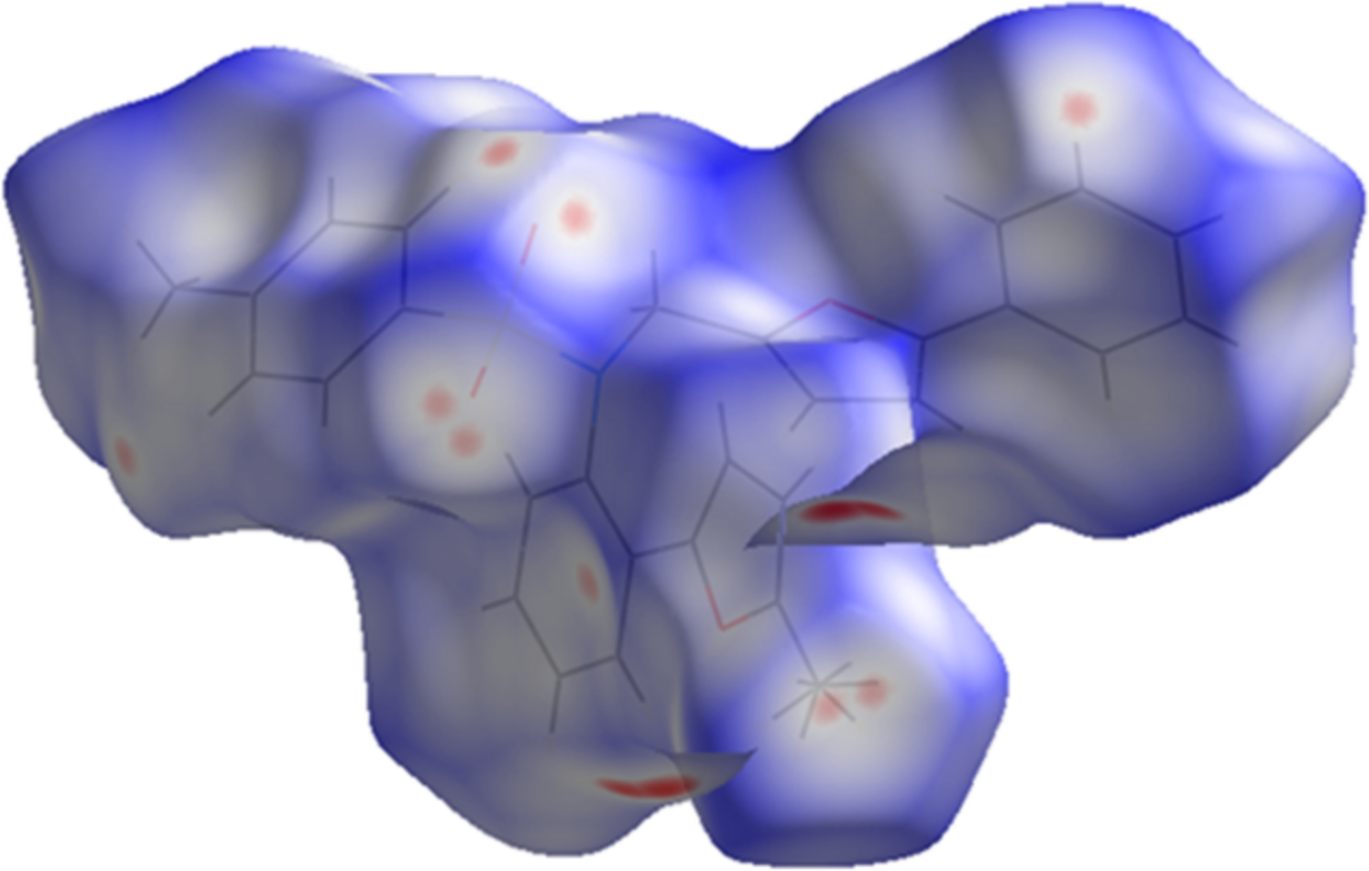
Hirshfeld surface of the title compound mapped with *d*_norm_.

**Figure 6:**
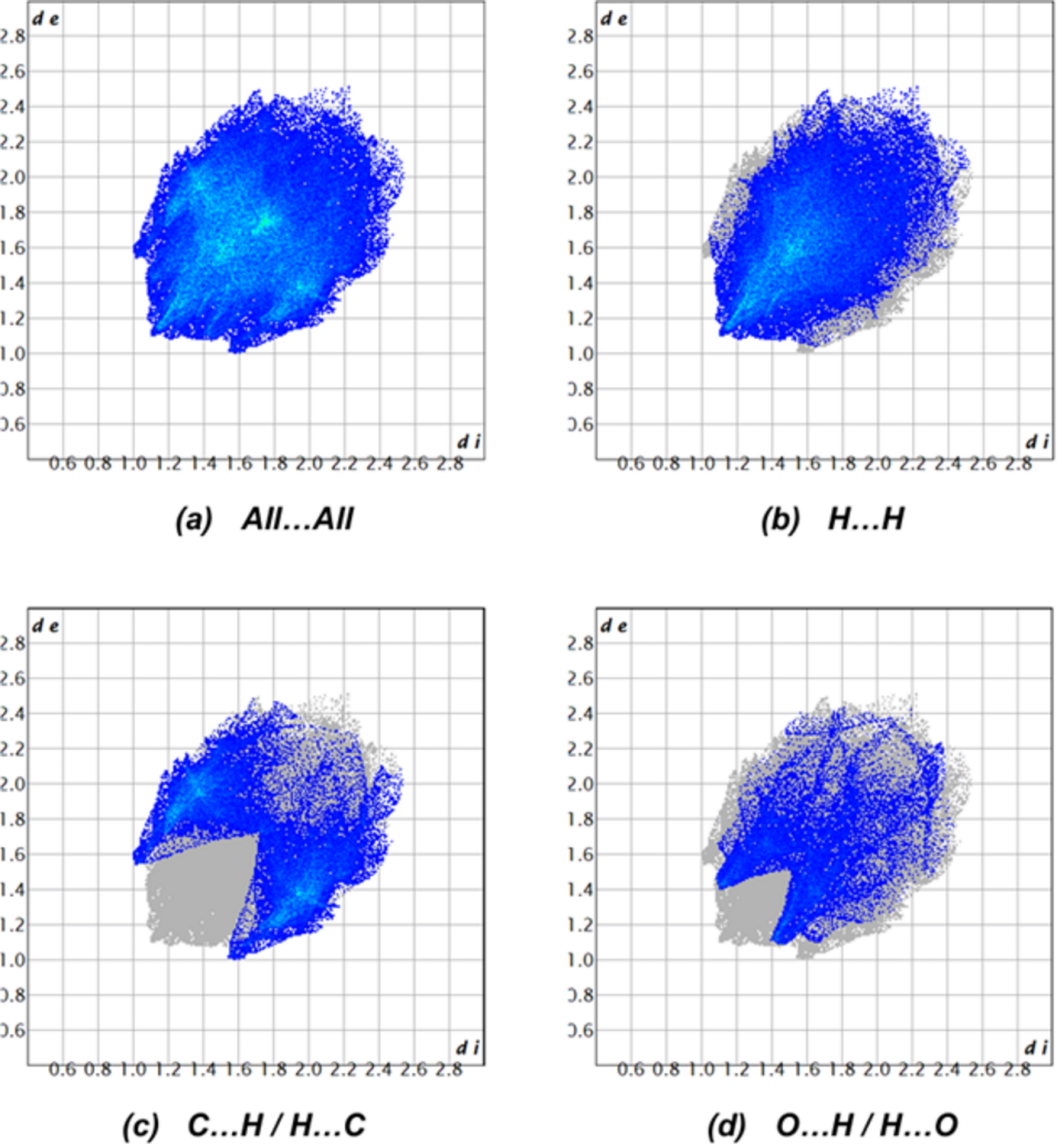
The two-dimensional fingerprint plots for the compound showing (*a*) all inter­actions, and delineated into (*b*) H⋯H (53.2%), (*c*) C⋯H/H⋯C (28.9%) and (*d*) O⋯H/H⋯O (13.8%) inter­actions. The *d*_i_ and *d*_e_ values are the closest inter­nal and external distances (in Å) from given points on the Hirshfeld surface.

A Hirshfeld surface analysis was carried out using *Crystal Explorer 17.5* (Spackman *et al.*, 2021 ▸) to investigate the inter­molecular inter­actions (Tables 1 ▸ and 2 ▸) in the crystal of the compound. The Hirshfeld surface mapped with the *d*_norm_ function over the range −0.1546 to 1. 3220 a.u. (Fig. 5 ▸) illustrates contact distances that are equal, shorter, and longer in relation to the sum of van der Waals radii, represented by white, red, and blue colors, respectively, with bright-red spots indicating the corresponding donors and acceptors. According to the two-dimensional fingerprint plots, the inter­molecular H⋯H, C⋯H/H⋯C and O⋯H/H⋯O contacts make the most important contributions to the Hirshfeld surface of 53.2%, 28.9% and 13.8%, respectively (Fig. 6 ▸). Smaller contributions are made by O⋯C/C⋯O (2.4%), C⋯C (1.6%) and O⋯O (0.1%) inter­actions.

## 4. Database survey

A search of the Cambridge Structural Database (CSD, version 6.00, update April 2025; Groom *et al.*, 2016 ▸) for the 2-(furan-2-yl)-*N*-[(furan-2-yl) meth­yl]aniline unit gave four hits, *viz*. CSD refcodes IFUBOB (Zubkov *et al.*, 2008*a* ▸), LOKWOB (Burkin *et al.*, 2024 ▸), SEBWIG (Zubkov *et al.*, 2006 ▸) and VOCWAM (Zubkov *et al.*, 2008*b* ▸).

IFUBOB, LOKWOB and VOCWAM crystallize in the triclinic *P*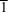 space group, and SEBWIG in the monoclinic *P*2_1_/*c* space group like the title compound. While in the title compound inter­molecular C—H⋯O hydrogen bonds, C—H..π and π–π inter­actions are observed, in IFUBOB, LOKWOB, SEBWIG and VOCWAM, the mol­ecules are linked by C—H⋯O hydrogen bonds, forming a three-dimensional network. C—H⋯π inter­actions were also observed in all except VOCWAM.

In addition, four related compounds containing the O=S=O group are HUSFIO (Burkin *et al.*, 2025 ▸), YIKROD (Mammadova *et al.*, 2023*a* ▸), KETGID (Schinke *et al.*, 2022 ▸) and LUJKUA (Vinaya *et al.*, 2024 ▸). In the crystal of HUSFIO, the mol­ecules form sheets parallel to the (002) plane due to C—H⋯O and C—H⋯F inter­actions. In addition, the mol­ecules are connected in the *a*-axis direction by S—O⋯π and π–π inter­actions, and there are van der Waals inter­actions between the mol­ecular sheets. In YIKROD, mol­ecules are connected *via* C—H⋯O and C—H⋯N hydrogen bonds, forming layers parallel to the (100) plane. These layers are inter­connected by C⋯H inter­actions and weak van der Waals inter­actions. In KETGID, the crystal structure features three short inter­molecular C—H⋯O contacts involving the methane­sulfonyl-O atoms. In LUJKUA, the asymmetric unit contains two distinct mol­ecules, which exhibit differences in conformation resulting from a variation in key torsion angles. These distinctions influence the mol­ecular orientation and inter­molecular inter­actions, with strong N—H⋯N and N—H⋯O hydrogen bonds forming a centrosymmetric tetra­mer stabilized by π–π stacking.

## 5. Synthesis and crystallization

**Figure 7:**
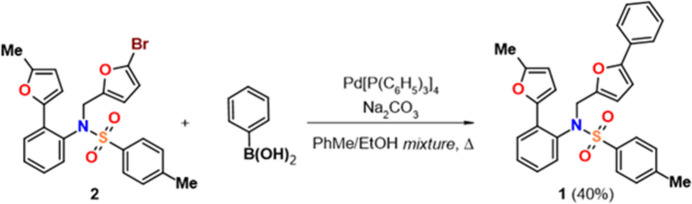
Synthesis of 4-methyl-*N*-[2-(5-methyl-2-fur­yl) phen­yl]-*N*-[(5-phenyl-2-fur­yl) meth­yl]benzene­sulfonamide.

A 125 mL dry Schlenk tube was charged with *N*-[(5-bromo­furan-2-yl)meth­yl]-4-methyl-*N*-[2-(5-methyl­furan-2­yl)phen­yl]benzene-1-sulfonamide (500 mg, 1.03 mmol) in a mixture of ethanol/toluene (10 mL, 1:1). To the reaction mixture in the presence of a 2 *M* water solution of Na_2_CO_3_ (5.38 mmol, 2.71 mL), phenyl­boronic acid (250 mg, 2.06 mmol) was added (Fig. 7 ▸). Argon was bubbled through the solution for 10 min. Then tetra­kis­(tri­phenyl­phosphine)palladium (59.3 mg, 51.4 µmol) was added in a gentle flow of argon. The reaction mixture was stirred at 383 K for 5 h. After the cooling of the reaction to room temperature, the resulting mixture was treated with water (30 mL) and extracted with EtOAc (3 × 10 mL), and treated in a usual manner to give a solid that was purified by silica gel column chromatography (eluent: heptane to hepta­ne/ethyl ­acetate, 10:1). The title compound was obtained as a light-brown solid, yield 40%, 199.2 mg (0.41 mmol); m.p. 456 K. A single- crystal of the compound was grown from a hepta­ne/ethyl acetate mixture. IR (KBr), *ν* (cm^−1^): 1348 (ν_as_ SO_2_), 1165 (ν_s_ SO_2_). ^1^H NMR (700.2 MHz, CDCl_3_) (*J*, Hz): *δ* 7.84 (*d*, *J* = 7.9, 1H, H Ar), 7.69 (*d*, *J* = 8.1, 2H, H Ar), 7.39 (*d*, *J* = 7.6, 2H, H Ar), 7.34–7.31 (*m*, 3H, H Ar), 7.25–7.23 (*m*, 3H, H Ar), 7.08 (*d*, *J* = 3.1, 1H, H Fur), 7.01 (*t*, *J* = 7.6, 1H, H Ar), 6.65 (*d*, *J* = 7.9, 1H, H Ar), 6.39 (*d*, *J* = 3.3, 1H, H Fur), 6.08 (*d*, *J* = 3.1, 1H, H Fur), 6.03 (*d*, *J* = 3.3, 1H, H Fur), 5.01 (*d*, *J* = 15.5, 1H, NC*H*), 4.65 (*d*, *J* = 15.5, 1H, NC*H*), 2.39 (*s*, 3H, CH_3_), 2.32 (*s*, 3H, CH_3_). ^13^C{^1^H} NMR (176.1 MHz, CDCl_3_): *δ* 153.9, 152.0, 148.9, 148.4, 143.3, 137.4, 133.9, 131.8, 130.5, 130.3, 129.4 (2C), 128.8, 128.5 (2C), 128.0 (2C), 127.4, 127.1, 126.7, 123.7 (2C), 112.2, 111.4, 108.4, 105.5, 47.4, 21.5, 13.7. MS (ESI) *m*/*z*: [*M* + H]^+^ 484. Elemental analysis calculated (%) for C_29_H_25_NO_4_S: C 72.03, H 5.21, N 2.90, S 6.63; found: C 71.89, H 5.48, N 3.18, S 6.33.

## 6. Refinement

Crystal data, data collection and structure refinement details are summarized in Table 3 ▸. All C-bound H atoms were positioned geometrically (C—H = 0.95 and 0.99 Å) and refined using a riding model with *U*_iso_(H) = 1.2 or 1.5*U*_eq_(C). One of the methyl groups (C22) was found to be disordered; it was treated as an idealized disordered methyl group, with two positions rotated from each other by 60°, and the site-occupation factors were fixed at 0.5.

## Supplementary Material

Crystal structure: contains datablock(s) I. DOI: 10.1107/S2056989025006231/ee2017sup1.cif

Structure factors: contains datablock(s) I. DOI: 10.1107/S2056989025006231/ee2017Isup2.hkl

Supporting information file. DOI: 10.1107/S2056989025006231/ee2017Isup3.cml

CCDC reference: 2472590

Additional supporting information:  crystallographic information; 3D view; checkCIF report

## Figures and Tables

**Figure 3:**
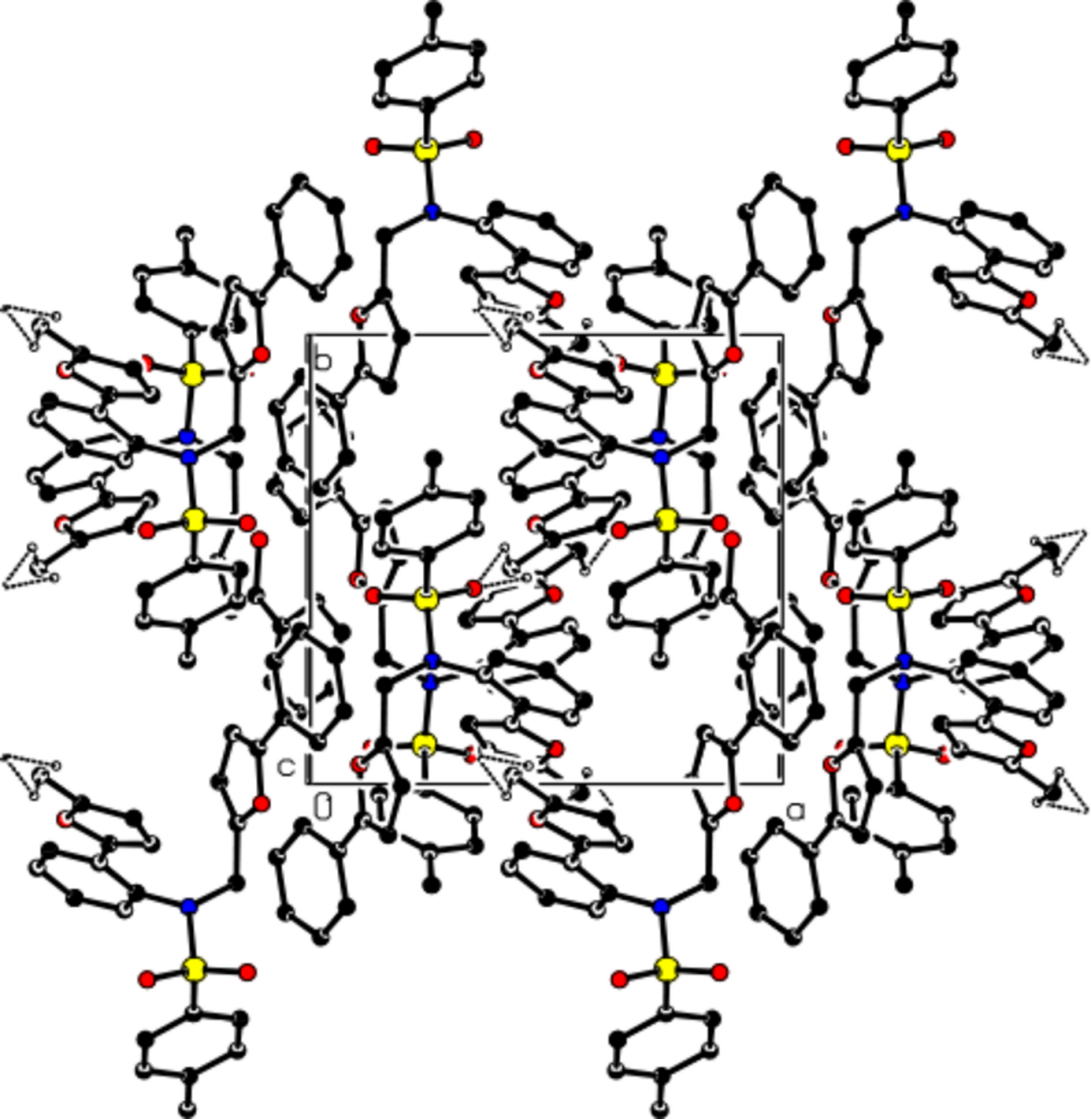
A view of the mol­ecular packing along the *c* axis, showing the C—H⋯O inter­actions.

**Table 1:** Hydrogen-bond geometry (Å, °) *Cg*3 and *C*g5 are the centroids of the C6–C12 and C23–C28 rings, respectively.

*D*—H⋯*A*	*D*—H	H⋯*A*	*D*⋯*A*	*D*—H⋯*A*
C1—H1*B*⋯O4	0.99	2.43	2.9091 (16)	109
C14—H14⋯O2	0.95	2.36	2.7265 (16)	102
C19—H19⋯N1	0.95	2.56	3.0211 (16)	110
C22—H22*C*⋯O3^i^	0.98	2.60	3.2636 (18)	125
C22—H22*D*⋯O3^i^	0.98	2.60	3.2636 (18)	126
C24—H24⋯O3	0.95	2.55	2.9196 (16)	104
C1—H1*A*⋯*Cg*3^ii^	0.99	2.94	3.6930 (14)	134
C4—H4⋯*Cg*5^iii^	0.95	2.86	3.7996 (14)	172
C29—H29*C*⋯*Cg*3^iv^	0.98	2.97	3.6676 (17)	129

Symmetry codes: (i) 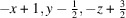; (ii) 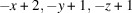; (iii) 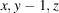; (iv) 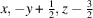.

**Table 2:** Summary of short inter­atomic contacts (Å)

Contact	Distance	Symmetry operation
H22*A*⋯H3	2.55	1 − *x*, 1 − *y*, 1 − *z*
H29*B*⋯H22*E*	2.49	1 − *x*, 2 − *y*, 1 − *z*
O3⋯H22*D*	2.60	1 − *x*, 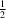 + *y*, 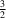 − *z*
O4⋯H10	2.60	*x*, 1 + *y*, *z*
H19⋯H9	2.47	2 − *x*, 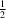 + *y*, 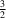 − *z*
H28⋯H28	2.40	2 − *x*, 2 − *y*, 1 − *z*
H27⋯H10	2.55	2 − *x*, 1 − *y*, 1 − *z*
H22C⋯H16	2.43	*x*, 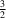 − *y*, 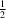 + *z*
H10⋯O4	2.60	*x*, −1 + *y*, *z*
H22*D*⋯O3	2.60	1 − *x*, − 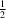 + *y*, 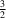 − *z*
H16⋯H29*B*	2.45	1 − *x*, − 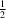 + *y*, 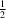 − *z*

**Table 3:** Experimental details

Crystal data	
Chemical formula	C_29_H_25_NO_4_S
*M*_r_	483.56
Crystal system, space group	Monoclinic, *P*2_1_/*c*
Temperature (K)	100
*a*, *b*, *c* (Å)	11.71270 (9), 11.10322 (9), 18.30473 (13)
β (°)	93.6993 (7)
*V* (Å^3^)	2375.55 (3)
*Z*	4
Radiation type	Cu *K*α
μ (mm^−1^)	1.51
Crystal size (mm)	0.21 × 0.15 × 0.12

Data collection	
Diffractometer	Rigaku XtaLAB Synergy-S, HyPix-6000HE area-detector
Absorption correction	Multi-scan (*CrysAlis PRO*; Rigaku OD, 2021 ▸)
*T*_min_, *T*_max_	0.615, 1.000
No. of measured, independent and observed [*I* > 2σ(*I*)] reflections	28196, 5133, 4843
*R*_int_	0.039
(sin θ/λ)_max_ (Å^−1^)	0.639

Refinement	
*R*[*F*^2^ > 2σ(*F*^2^)], *wR*(*F*^2^), *S*	0.038, 0.103, 1.07
No. of reflections	5133
No. of parameters	318
H-atom treatment	H-atom parameters constrained
Δρ_max_, Δρ_min_ (e Å^−3^)	0.36, −0.48

Computer programs: *CrysAlis PRO* (Rigaku OD, 2021 ▸), *SHELXT* (Sheldrick, 2015*a* ▸), *SHELXL* (Sheldrick, 2015*b* ▸), *ORTEP-3 for Windows* (Farrugia, 2012 ▸) and *PLATON* (Spek, 2020 ▸).

## supplementary crystallographic information

### 4-Methyl-N-[2-(5-methylfuran-2-yl)phenyl]-N-[(5-phenylfuran-2-yl)methyl]benzenesulfonamide Crystal data

**Table:**

C_29_H_25_NO_4_S	*F*(000) = 1016
*M**_r_* = 483.56	*D*_x_ = 1.352 Mg m^−^^3^
Monoclinic, *P*2_1_/*c*	Cu *K*α radiation, λ = 1.54184 Å
*a* = 11.71270 (9) Å	Cell parameters from 18959 reflections
*b* = 11.10322 (9) Å	θ = 3.8–79.3°
*c* = 18.30473 (13) Å	µ = 1.51 mm^−^^1^
β = 93.6993 (7)°	*T* = 100 K
*V* = 2375.55 (3) Å^3^	Prism, colourless
*Z* = 4	0.21 × 0.15 × 0.12 mm

### 4-Methyl-N-[2-(5-methylfuran-2-yl)phenyl]-N-[(5-phenylfuran-2-yl)methyl]benzenesulfonamide Data collection

**Table:**

Rigaku XtaLAB Synergy-S, HyPix-6000HE area-detector diffractometer	4843 reflections with *I* > 2σ(*I*)
Radiation source: micro-focus sealed X-ray tube	*R*_int_ = 0.039
φ and ω scans	θ_max_ = 80.0°, θ_min_ = 3.8°
Absorption correction: multi-scan (CrysAlisPro; Rigaku OD, 2021)	*h* = −14→14
*T*_min_ = 0.615, *T*_max_ = 1.000	*k* = −14→14
28196 measured reflections	*l* = −19→23
5133 independent reflections	

### 4-Methyl-N-[2-(5-methylfuran-2-yl)phenyl]-N-[(5-phenylfuran-2-yl)methyl]benzenesulfonamide Refinement

**Table:**

Refinement on *F*^2^	Hydrogen site location: inferred from neighbouring sites
Least-squares matrix: full	H-atom parameters constrained
*R*[*F*^2^ > 2σ(*F*^2^)] = 0.038	*w* = 1/[σ^2^(*F*_o_^2^) + (0.0554*P*)^2^ + 0.936*P*] where *P* = (*F*_o_^2^ + 2*F*_c_^2^)/3
*wR*(*F*^2^) = 0.103	(Δ/σ)_max_ = 0.002
*S* = 1.07	Δρ_max_ = 0.36 e Å^−^^3^
5133 reflections	Δρ_min_ = −0.48 e Å^−^^3^
318 parameters	Extinction correction: SHELXL-2019/2 (Sheldrick, 2015b), Fc^*^=kFc[1+0.001xFc^2^λ^3^/sin(2θ)]^-1/4^
0 restraints	Extinction coefficient: 0.00047 (13)

### 4-Methyl-N-[2-(5-methylfuran-2-yl)phenyl]-N-[(5-phenylfuran-2-yl)methyl]benzenesulfonamide Special details

**Table:**

Experimental. CrysAlisPro 1.171.43.129a (Rigaku OD, 2021). Empirical absorption correction using spherical harmonics, implemented in SCALE3 ABSPACK scaling algorithm.
Geometry. All esds (except the esd in the dihedral angle between two l.s. planes) are estimated using the full covariance matrix. The cell esds are taken into account individually in the estimation of esds in distances, angles and torsion angles; correlations between esds in cell parameters are only used when they are defined by crystal symmetry. An approximate (isotropic) treatment of cell esds is used for estimating esds involving l.s. planes.

### 4-Methyl-N-[2-(5-methylfuran-2-yl)phenyl]-N-[(5-phenylfuran-2-yl)methyl]benzenesulfonamide Fractional atomic coordinates and isotropic or equivalent isotropic displacement parameters (Å^2^)

**Table:**

	*x*	*y*	*z*	*U*_iso_*/*U*_eq_	Occ. (<1)
S1	0.75420 (3)	0.91249 (3)	0.55524 (2)	0.01882 (10)	
O1	0.89563 (7)	0.54353 (8)	0.56336 (5)	0.01940 (19)	
O2	0.48043 (8)	0.57815 (8)	0.62957 (5)	0.0216 (2)	
O3	0.65373 (9)	0.93517 (9)	0.59358 (5)	0.0259 (2)	
O4	0.86552 (9)	0.91925 (9)	0.59281 (5)	0.0266 (2)	
N1	0.74228 (9)	0.77377 (9)	0.52304 (5)	0.0174 (2)	
C1	0.84333 (10)	0.72008 (12)	0.48961 (7)	0.0200 (2)	
H1A	0.840447	0.740049	0.436818	0.024*	
H1B	0.914364	0.754977	0.513030	0.024*	
C2	0.84531 (10)	0.58720 (12)	0.49885 (7)	0.0197 (3)	
C3	0.80749 (11)	0.49497 (12)	0.45546 (7)	0.0220 (3)	
H3	0.769646	0.501157	0.408125	0.026*	
C4	0.83540 (11)	0.38660 (12)	0.49453 (7)	0.0213 (3)	
H4	0.819986	0.306638	0.478271	0.026*	
C5	0.88842 (10)	0.42029 (11)	0.55961 (7)	0.0191 (2)	
C6	0.94092 (10)	0.35150 (11)	0.62083 (7)	0.0193 (2)	
C7	1.01454 (12)	0.40632 (12)	0.67457 (7)	0.0221 (3)	
H7	1.027068	0.490801	0.673127	0.027*	
C8	1.06915 (12)	0.33764 (13)	0.72983 (7)	0.0266 (3)	
H8	1.119454	0.375271	0.765674	0.032*	
C9	1.05054 (13)	0.21398 (14)	0.73295 (8)	0.0282 (3)	
H9	1.088203	0.167234	0.770716	0.034*	
C10	0.97662 (12)	0.15918 (12)	0.68057 (7)	0.0256 (3)	
H10	0.963472	0.074865	0.682719	0.031*	
C11	0.92192 (11)	0.22725 (12)	0.62513 (7)	0.0217 (3)	
H11	0.871108	0.189155	0.589761	0.026*	
C12	0.63199 (10)	0.74259 (11)	0.48753 (6)	0.0167 (2)	
C13	0.55292 (10)	0.67513 (11)	0.52494 (6)	0.0172 (2)	
C14	0.44821 (11)	0.64726 (11)	0.48680 (7)	0.0202 (2)	
H14	0.393701	0.600160	0.510350	0.024*	
C15	0.42232 (11)	0.68661 (12)	0.41580 (7)	0.0225 (3)	
H15	0.350198	0.667796	0.391789	0.027*	
C16	0.50147 (11)	0.75340 (12)	0.37972 (7)	0.0222 (3)	
H16	0.483966	0.780739	0.331103	0.027*	
C17	0.60674 (11)	0.77981 (11)	0.41561 (7)	0.0197 (2)	
H17	0.662090	0.823758	0.390805	0.024*	
C18	0.57319 (11)	0.63322 (11)	0.60056 (7)	0.0191 (2)	
C19	0.66248 (12)	0.63388 (13)	0.65182 (7)	0.0249 (3)	
H19	0.736417	0.666552	0.646406	0.030*	
C20	0.62372 (13)	0.57591 (13)	0.71549 (8)	0.0279 (3)	
H20	0.667186	0.562507	0.760376	0.033*	
C21	0.51367 (12)	0.54384 (12)	0.69972 (7)	0.0247 (3)	
C22	0.42627 (14)	0.48056 (14)	0.74114 (8)	0.0320 (3)	
H22A	0.354790	0.474037	0.710479	0.048*	0.5
H22B	0.453939	0.399773	0.754648	0.048*	0.5
H22C	0.412534	0.526204	0.785545	0.048*	0.5
H22D	0.459386	0.459306	0.789969	0.048*	0.5
H22E	0.360236	0.533570	0.745800	0.048*	0.5
H22F	0.401641	0.407138	0.714903	0.048*	0.5
C23	0.75261 (11)	1.01057 (11)	0.47944 (6)	0.0181 (2)	
C24	0.65102 (11)	1.06855 (12)	0.45602 (7)	0.0203 (2)	
H24	0.584841	1.061068	0.483077	0.024*	
C25	0.64818 (11)	1.13728 (11)	0.39262 (7)	0.0216 (3)	
H25	0.579097	1.176375	0.376150	0.026*	
C26	0.74469 (11)	1.15002 (11)	0.35268 (7)	0.0212 (3)	
C27	0.84635 (11)	1.09300 (12)	0.37789 (7)	0.0215 (3)	
H27	0.913159	1.102499	0.351658	0.026*	
C28	0.85073 (10)	1.02279 (12)	0.44071 (7)	0.0201 (2)	
H28	0.919719	0.983500	0.457156	0.024*	
C29	0.74108 (14)	1.22441 (14)	0.28389 (8)	0.0319 (3)	
H29A	0.784934	1.298663	0.292996	0.048*	
H29B	0.661506	1.244615	0.269002	0.048*	
H29C	0.774435	1.178402	0.244843	0.048*	

### 4-Methyl-N-[2-(5-methylfuran-2-yl)phenyl]-N-[(5-phenylfuran-2-yl)methyl]benzenesulfonamide Atomic displacement parameters (Å^2^)

**Table:**

	*U*^11^	*U*^22^	*U*^33^	*U*^12^	*U*^13^	*U*^23^
S1	0.02193 (17)	0.01953 (16)	0.01493 (16)	−0.00240 (10)	0.00068 (11)	−0.00034 (10)
O1	0.0181 (4)	0.0180 (4)	0.0221 (4)	0.0004 (3)	0.0010 (3)	0.0003 (3)
O2	0.0232 (4)	0.0213 (4)	0.0210 (4)	0.0005 (3)	0.0066 (3)	0.0044 (3)
O3	0.0330 (5)	0.0248 (5)	0.0210 (5)	−0.0009 (4)	0.0098 (4)	−0.0027 (4)
O4	0.0294 (5)	0.0283 (5)	0.0209 (5)	−0.0062 (4)	−0.0081 (4)	0.0019 (3)
N1	0.0158 (5)	0.0180 (5)	0.0184 (5)	−0.0002 (4)	0.0015 (4)	0.0006 (4)
C1	0.0155 (5)	0.0220 (6)	0.0230 (6)	0.0005 (4)	0.0040 (4)	0.0025 (5)
C2	0.0152 (5)	0.0231 (6)	0.0211 (6)	0.0014 (4)	0.0029 (4)	0.0026 (4)
C3	0.0183 (6)	0.0255 (7)	0.0221 (6)	0.0000 (5)	0.0006 (4)	0.0005 (5)
C4	0.0186 (6)	0.0218 (6)	0.0235 (6)	−0.0011 (5)	0.0011 (5)	−0.0022 (5)
C5	0.0161 (5)	0.0185 (6)	0.0230 (6)	−0.0004 (4)	0.0042 (4)	−0.0010 (4)
C6	0.0177 (5)	0.0206 (6)	0.0198 (6)	0.0009 (4)	0.0038 (4)	−0.0013 (5)
C7	0.0247 (6)	0.0203 (6)	0.0215 (6)	−0.0001 (5)	0.0022 (5)	−0.0023 (5)
C8	0.0281 (6)	0.0295 (7)	0.0216 (6)	−0.0010 (5)	−0.0020 (5)	−0.0020 (5)
C9	0.0310 (7)	0.0285 (7)	0.0247 (6)	0.0043 (6)	−0.0014 (5)	0.0043 (5)
C10	0.0298 (7)	0.0196 (6)	0.0277 (7)	0.0020 (5)	0.0033 (5)	0.0015 (5)
C11	0.0221 (6)	0.0206 (6)	0.0225 (6)	−0.0006 (5)	0.0024 (5)	−0.0019 (5)
C12	0.0169 (5)	0.0154 (5)	0.0180 (5)	0.0017 (4)	0.0015 (4)	−0.0015 (4)
C13	0.0175 (5)	0.0158 (5)	0.0184 (6)	0.0031 (4)	0.0024 (4)	−0.0004 (4)
C14	0.0176 (6)	0.0196 (6)	0.0235 (6)	0.0002 (4)	0.0028 (4)	−0.0005 (5)
C15	0.0199 (6)	0.0238 (6)	0.0234 (6)	0.0002 (5)	−0.0030 (5)	−0.0020 (5)
C16	0.0264 (6)	0.0224 (6)	0.0173 (6)	0.0000 (5)	−0.0028 (5)	0.0007 (5)
C17	0.0219 (6)	0.0188 (6)	0.0184 (6)	−0.0009 (5)	0.0021 (4)	0.0005 (4)
C18	0.0200 (6)	0.0175 (6)	0.0203 (6)	0.0009 (4)	0.0055 (4)	0.0017 (4)
C19	0.0257 (6)	0.0288 (7)	0.0202 (6)	−0.0007 (5)	0.0013 (5)	0.0045 (5)
C20	0.0361 (8)	0.0299 (7)	0.0178 (6)	0.0025 (6)	0.0027 (5)	0.0048 (5)
C21	0.0336 (7)	0.0217 (6)	0.0197 (6)	0.0046 (5)	0.0082 (5)	0.0035 (5)
C22	0.0402 (8)	0.0282 (7)	0.0293 (7)	0.0036 (6)	0.0157 (6)	0.0080 (6)
C23	0.0204 (6)	0.0163 (5)	0.0175 (5)	−0.0013 (4)	0.0002 (4)	−0.0018 (4)
C24	0.0191 (6)	0.0191 (6)	0.0228 (6)	0.0006 (4)	0.0028 (4)	−0.0029 (5)
C25	0.0209 (6)	0.0189 (6)	0.0248 (6)	0.0035 (5)	−0.0010 (5)	−0.0024 (5)
C26	0.0265 (6)	0.0178 (6)	0.0192 (6)	0.0012 (5)	0.0002 (5)	−0.0014 (4)
C27	0.0204 (6)	0.0226 (6)	0.0220 (6)	0.0004 (5)	0.0044 (5)	0.0001 (5)
C28	0.0177 (6)	0.0207 (6)	0.0216 (6)	0.0002 (4)	−0.0006 (4)	−0.0002 (5)
C29	0.0374 (8)	0.0320 (8)	0.0263 (7)	0.0064 (6)	0.0031 (6)	0.0082 (6)

### 4-Methyl-N-[2-(5-methylfuran-2-yl)phenyl]-N-[(5-phenylfuran-2-yl)methyl]benzenesulfonamide Geometric parameters (Å, º)

**Table:**

S1—O3	1.4309 (10)	C14—C15	1.3860 (18)
S1—O4	1.4363 (10)	C14—H14	0.9500
S1—N1	1.6518 (11)	C15—C16	1.3873 (19)
S1—C23	1.7630 (13)	C15—H15	0.9500
O1—C5	1.3724 (15)	C16—C17	1.3905 (18)
O1—C2	1.3737 (15)	C16—H16	0.9500
O2—C21	1.3715 (16)	C17—H17	0.9500
O2—C18	1.3823 (15)	C18—C19	1.3593 (18)
N1—C12	1.4500 (15)	C19—C20	1.4307 (19)
N1—C1	1.4913 (15)	C19—H19	0.9500
C1—C2	1.4850 (18)	C20—C21	1.350 (2)
C1—H1A	0.9900	C20—H20	0.9500
C1—H1B	0.9900	C21—C22	1.4888 (19)
C2—C3	1.3530 (19)	C22—H22A	0.9800
C3—C4	1.4268 (18)	C22—H22B	0.9800
C3—H3	0.9500	C22—H22C	0.9800
C4—C5	1.3599 (18)	C22—H22D	0.9800
C4—H4	0.9500	C22—H22E	0.9800
C5—C6	1.4589 (18)	C22—H22F	0.9800
C6—C11	1.4004 (18)	C23—C28	1.3953 (17)
C6—C7	1.4044 (18)	C23—C24	1.3958 (18)
C7—C8	1.3894 (19)	C24—C25	1.3876 (19)
C7—H7	0.9500	C24—H24	0.9500
C8—C9	1.392 (2)	C25—C26	1.3924 (18)
C8—H8	0.9500	C25—H25	0.9500
C9—C10	1.390 (2)	C26—C27	1.4003 (18)
C9—H9	0.9500	C26—C29	1.5043 (18)
C10—C11	1.3884 (19)	C27—C28	1.3873 (18)
C10—H10	0.9500	C27—H27	0.9500
C11—H11	0.9500	C28—H28	0.9500
C12—C17	1.3933 (17)	C29—H29A	0.9800
C12—C13	1.4034 (17)	C29—H29B	0.9800
C13—C14	1.4060 (17)	C29—H29C	0.9800
C13—C18	1.4650 (17)		
			
O3—S1—O4	120.39 (6)	C13—C14—H14	119.1
O3—S1—N1	106.61 (6)	C14—C15—C16	120.16 (12)
O4—S1—N1	105.83 (6)	C14—C15—H15	119.9
O3—S1—C23	108.07 (6)	C16—C15—H15	119.9
O4—S1—C23	107.92 (6)	C15—C16—C17	119.16 (11)
N1—S1—C23	107.39 (5)	C15—C16—H16	120.4
C5—O1—C2	106.70 (10)	C17—C16—H16	120.4
C21—O2—C18	107.46 (10)	C16—C17—C12	120.77 (11)
C12—N1—C1	115.36 (10)	C16—C17—H17	119.6
C12—N1—S1	115.78 (8)	C12—C17—H17	119.6
C1—N1—S1	117.91 (8)	C19—C18—O2	109.02 (11)
C2—C1—N1	110.94 (10)	C19—C18—C13	136.14 (12)
C2—C1—H1A	109.5	O2—C18—C13	114.84 (11)
N1—C1—H1A	109.5	C18—C19—C20	106.82 (12)
C2—C1—H1B	109.5	C18—C19—H19	126.6
N1—C1—H1B	109.5	C20—C19—H19	126.6
H1A—C1—H1B	108.0	C21—C20—C19	107.05 (12)
C3—C2—O1	110.09 (11)	C21—C20—H20	126.5
C3—C2—C1	133.08 (12)	C19—C20—H20	126.5
O1—C2—C1	116.83 (11)	C20—C21—O2	109.65 (12)
C2—C3—C4	106.76 (11)	C20—C21—C22	133.93 (13)
C2—C3—H3	126.6	O2—C21—C22	116.42 (13)
C4—C3—H3	126.6	C21—C22—H22A	109.5
C5—C4—C3	106.50 (12)	C21—C22—H22B	109.5
C5—C4—H4	126.7	H22A—C22—H22B	109.5
C3—C4—H4	126.7	C21—C22—H22C	109.5
C4—C5—O1	109.95 (11)	H22A—C22—H22C	109.5
C4—C5—C6	132.45 (12)	H22B—C22—H22C	109.5
O1—C5—C6	117.52 (11)	H22D—C22—H22E	109.5
C11—C6—C7	118.70 (12)	H22D—C22—H22F	109.5
C11—C6—C5	119.89 (11)	H22E—C22—H22F	109.5
C7—C6—C5	121.36 (12)	C28—C23—C24	120.86 (12)
C8—C7—C6	120.33 (12)	C28—C23—S1	119.52 (10)
C8—C7—H7	119.8	C24—C23—S1	119.47 (10)
C6—C7—H7	119.8	C25—C24—C23	118.97 (12)
C7—C8—C9	120.36 (13)	C25—C24—H24	120.5
C7—C8—H8	119.8	C23—C24—H24	120.5
C9—C8—H8	119.8	C24—C25—C26	121.23 (12)
C10—C9—C8	119.71 (13)	C24—C25—H25	119.4
C10—C9—H9	120.1	C26—C25—H25	119.4
C8—C9—H9	120.1	C25—C26—C27	118.88 (12)
C11—C10—C9	120.23 (13)	C25—C26—C29	121.02 (12)
C11—C10—H10	119.9	C27—C26—C29	120.10 (12)
C9—C10—H10	119.9	C28—C27—C26	120.82 (12)
C10—C11—C6	120.65 (12)	C28—C27—H27	119.6
C10—C11—H11	119.7	C26—C27—H27	119.6
C6—C11—H11	119.7	C27—C28—C23	119.22 (12)
C17—C12—C13	120.85 (11)	C27—C28—H28	120.4
C17—C12—N1	118.77 (11)	C23—C28—H28	120.4
C13—C12—N1	120.37 (10)	C26—C29—H29A	109.5
C12—C13—C14	117.20 (11)	C26—C29—H29B	109.5
C12—C13—C18	123.97 (11)	H29A—C29—H29B	109.5
C14—C13—C18	118.83 (11)	C26—C29—H29C	109.5
C15—C14—C13	121.82 (12)	H29A—C29—H29C	109.5
C15—C14—H14	119.1	H29B—C29—H29C	109.5
			
O3—S1—N1—C12	45.99 (10)	N1—C12—C13—C18	0.86 (18)
O4—S1—N1—C12	175.28 (8)	C12—C13—C14—C15	−1.40 (18)
C23—S1—N1—C12	−69.63 (9)	C18—C13—C14—C15	178.37 (12)
O3—S1—N1—C1	−171.44 (9)	C13—C14—C15—C16	1.3 (2)
O4—S1—N1—C1	−42.15 (10)	C14—C15—C16—C17	0.2 (2)
C23—S1—N1—C1	72.93 (10)	C15—C16—C17—C12	−1.59 (19)
C12—N1—C1—C2	−66.81 (13)	C13—C12—C17—C16	1.51 (19)
S1—N1—C1—C2	150.47 (9)	N1—C12—C17—C16	−179.11 (11)
C5—O1—C2—C3	−0.08 (13)	C21—O2—C18—C19	−0.35 (14)
C5—O1—C2—C1	−179.58 (10)	C21—O2—C18—C13	179.55 (10)
N1—C1—C2—C3	95.11 (16)	C12—C13—C18—C19	−6.0 (2)
N1—C1—C2—O1	−85.53 (13)	C14—C13—C18—C19	174.20 (15)
O1—C2—C3—C4	−0.06 (14)	C12—C13—C18—O2	174.10 (11)
C1—C2—C3—C4	179.34 (13)	C14—C13—C18—O2	−5.66 (16)
C2—C3—C4—C5	0.17 (14)	O2—C18—C19—C20	0.37 (15)
C3—C4—C5—O1	−0.23 (14)	C13—C18—C19—C20	−179.50 (14)
C3—C4—C5—C6	−176.77 (13)	C18—C19—C20—C21	−0.25 (16)
C2—O1—C5—C4	0.19 (13)	C19—C20—C21—O2	0.04 (16)
C2—O1—C5—C6	177.31 (10)	C19—C20—C21—C22	179.25 (15)
C4—C5—C6—C11	−13.0 (2)	C18—O2—C21—C20	0.19 (15)
O1—C5—C6—C11	170.63 (11)	C18—O2—C21—C22	−179.18 (11)
C4—C5—C6—C7	164.31 (14)	O3—S1—C23—C28	168.18 (10)
O1—C5—C6—C7	−12.02 (17)	O4—S1—C23—C28	36.52 (12)
C11—C6—C7—C8	1.41 (19)	N1—S1—C23—C28	−77.17 (11)
C5—C6—C7—C8	−175.97 (12)	O3—S1—C23—C24	−16.16 (12)
C6—C7—C8—C9	−0.7 (2)	O4—S1—C23—C24	−147.82 (10)
C7—C8—C9—C10	−0.2 (2)	N1—S1—C23—C24	98.49 (11)
C8—C9—C10—C11	0.3 (2)	C28—C23—C24—C25	1.11 (19)
C9—C10—C11—C6	0.5 (2)	S1—C23—C24—C25	−174.49 (10)
C7—C6—C11—C10	−1.30 (19)	C23—C24—C25—C26	−0.57 (19)
C5—C6—C11—C10	176.12 (12)	C24—C25—C26—C27	−0.65 (19)
C1—N1—C12—C17	−63.82 (14)	C24—C25—C26—C29	179.86 (13)
S1—N1—C12—C17	79.71 (12)	C25—C26—C27—C28	1.35 (19)
C1—N1—C12—C13	115.57 (12)	C29—C26—C27—C28	−179.15 (13)
S1—N1—C12—C13	−100.90 (12)	C26—C27—C28—C23	−0.82 (19)
C17—C12—C13—C14	−0.01 (17)	C24—C23—C28—C27	−0.43 (19)
N1—C12—C13—C14	−179.38 (11)	S1—C23—C28—C27	175.17 (10)
C17—C12—C13—C18	−179.77 (11)		

### 4-Methyl-N-[2-(5-methylfuran-2-yl)phenyl]-N-[(5-phenylfuran-2-yl)methyl]benzenesulfonamide Hydrogen-bond geometry (Å, º)

*Cg*3 and *C*g5 are the centroids of the C6–C12 and C23–C28 rings, respectively.

**Table:**

*D*—H···*A*	*D*—H	H···*A*	*D*···*A*	*D*—H···*A*
C1—H1*B*···O4	0.99	2.43	2.9091 (16)	109
C14—H14···O2	0.95	2.36	2.7265 (16)	102
C19—H19···N1	0.95	2.56	3.0211 (16)	110
C22—H22*C*···O3^i^	0.98	2.60	3.2636 (18)	125
C22—H22*D*···O3^i^	0.98	2.60	3.2636 (18)	126
C24—H24···O3	0.95	2.55	2.9196 (16)	104
C1—H1*A*···*Cg*3^ii^	0.99	2.94	3.6930 (14)	134
C4—H4···*Cg*5^iii^	0.95	2.86	3.7996 (14)	172
C29—H29*C*···*Cg*3^iv^	0.98	2.97	3.6676 (17)	129

Symmetry codes: (i) −*x*+1, *y*−1/2, −*z*+3/2; (ii) −*x*+2, −*y*+1, −*z*+1; (iii) *x*, *y*−1, *z*; (iv) *x*, −*y*+1/2, *z*−3/2.
